# A longitudinal investigation of pragmatic language across contexts in autism and related neurodevelopmental conditions

**DOI:** 10.3389/fneur.2023.1155691

**Published:** 2023-07-21

**Authors:** Gary E. Martin, Michelle Lee, Klinton Bicknell, Adam Goodkind, Nell Maltman, Molly Losh

**Affiliations:** ^1^Department of Communication Sciences and Disorders, St. John’s University, Staten Island, NY, United States; ^2^Roxelyn and Richard Pepper Department of Communication Sciences and Disorders, Northwestern University, Evanston, IL, United States; ^3^Department of Child and Adolescent Psychiatry, Child Study Center, Hassenfeld Children’s Hospital at NYU Langone, New York, NY, United States; ^4^Department of Linguistics, Northwestern University, Evanston, IL, United States; ^5^Duolingo, Pittsburgh, PA, United States; ^6^Department of Communication Studies, Northwestern University, Evanston, IL, United States; ^7^Waisman Center, University of Wisconsin-Madison, Madison, WI, United States

**Keywords:** pragmatic language, social communication, autism spectrum disorder, fragile X syndrome, Down syndrome, longitudinal

## Abstract

**Background:**

Pragmatic language, or the use of language in social contexts, is a critical skill in daily life, supporting social interactions and the development of meaningful social relationships. Pragmatic language is universally impacted in autism spectrum disorder (ASD) and pragmatic deficits are also common in other neurodevelopmental conditions, particularly those related to ASD, such as fragile X syndrome (FXS). This study used a multi-method, longitudinal approach to characterize potentially unique pragmatic profiles across different neurodevelopmental disabilities, and across contexts that varied in degree of social demand. The utility of computational linguistic analyses, as an efficient tool for capturing pragmatic abilities, was also explored.

**Methods:**

Pragmatic skills of boys with idiopathic ASD (ASD-O, *n* = 43), FXS with and without ASD (FXS-ASD, *n* = 57; FXS-O, *n* = 14), Down syndrome (DS, *n* = 22), and typical development (TD, *n* = 24) were compared using variables obtained from a standardized measure, narrative, and semi-naturalistic conversation at up to three time points.

**Results:**

Pragmatic language was most significantly impacted among males with ASD-O and FXS-ASD across all three contexts, with more difficulties in the least structured context (conversation), and also some differences based on FXS comorbidity. Patterns of group differences were more nuanced for boys with FXS-O and DS, with context having less of an impact. Clinical groups demonstrated minimal changes in pragmatic skills with age, with some exceptions. Computational language measurement tools showed some utility for measuring pragmatic skills, but were not as successful as traditional methods at capturing differences between clinical groups.

**Conclusion:**

Overlap and differences between ASD and other forms of neurodevelopmental disability in general, and between idiopathic and syndromic ASD in particular, have important implications for developing precisely tailored assessment and intervention approaches, consistent with a personalized medicine approach to clinical study and care in ASD.

## 1. Introduction

Autism spectrum disorder (ASD) is characterized by a highly heterogeneous clinical presentation with core impairments in social communication and the presence of atypical restricted and repetitive behaviors (1). Pragmatic language, which refers to how language is used in social situations, is a critical skill in daily life, supporting social interactions and the development of meaningful social relationships. Pragmatic skills are impacted in ASD in ways that can interfere with successful communication and social interaction. Pragmatics are often impacted in other neurodevelopmental conditions as well, including those with etiologic connections to ASD, such as fragile X syndrome (FXS), the most common known single-gene cause of ASD. Understanding whether pragmatic skills are impacted in qualitatively similar ways across such conditions (and in idiopathic and syndromic ASD in particular) may facilitate the translation of targeted interventions across conditions. Tailoring assessment and treatment approaches based on a particular symptom profile or etiology, referred to as precision or personalized medicine, has emerged as a promising approach to clinical research and practice in ASD (2).

A strong genetic etiology of ASD is well established, with multiple risk genes identified, and high heritability observed, along with evidence of subclinical ASD-related phenotypes aggregating in first-degree relatives of individuals with ASD (3–7); however, ASD etiology remains highly complex, with multiple etiologic pathways identified [see (8) for review]. FXS is the most common single-gene disorder associated with ASD, with 60–74% of males with FXS meeting full diagnostic criteria for ASD (9–13), and more (up to 90%) demonstrating symptoms consistent with ASD (11, 14–21). FXS is caused by a Cytosine-Guanine-Guanine (CGG) repeat expansion of over 200 on the 5′ untranslated region of the Fragile X messenger ribonucleoprotein 1 gene (*FMR1*) on the X chromosome, inhibiting the production of Fragile X messenger ribonucleoprotein 1 (FMRP), an essential protein for several aspects of brain development (22, 23). *FMR1* regulates multiple known ASD risk genes (24–28), suggesting that studying ASD in the context of FXS may help to clarify genetic pathways related to ASD phenotypes.

Pragmatic language has emerged as a key phenotypic dimension showing overlap across ASD and FXS. Several studies suggest overlap in both the degree of pragmatic impairment and the specific quality of pragmatic challenges in individuals with idiopathic ASD (ASD-Only or ASD-O) and those with FXS who also meet criteria for ASD (FXS-ASD) (29–32). Additionally, qualitatively similar differences in pragmatic language have also been observed in first degree relatives of individuals with ASD (5, 33–36) and carriers of the *FMR1* premutation (5, 37), providing further evidence that overlapping pragmatic impairments in these groups may index common genetic influence related to *FMR1*.

Equally relevant to genetic and clinical endeavors, and to the personalized medicine approach in particular, is evidence of some important distinctions in the pragmatic phenotypes of FXS-ASD and ASD-O. Whereas cross-population comparisons of ASD-O and FXS-ASD have demonstrated a number of key similarities [e.g., difficulty with topic maintenance and elaboration, perseveration (29, 30)], unique patterns of differences are evident in conversational initiations and responsiveness, and aspects of conversational repair (29, 38), where individuals with ASD-O appear to have more profound difficulties or different types of difficulty than those with FXS-ASD. Therefore, delineating the specific pragmatic language profiles of individuals with ASD-O and FXS-ASD is necessary to identify specific characteristics that may be utilized in future research aimed more directly at understanding the role of *FMR1* variation in pragmatic language features, and to guide assessment and intervention approaches tailored to pragmatic profiles that are specific to a particular group. Further, understanding how such profiles may differ at different developmental periods, and be impacted by general cognitive-developmental factors is of critical importance.

This study examined longitudinal assessments of pragmatic language to characterize pragmatic profiles across boys with ASD-O, FXS-ASD, FXS-Only (FXS-O), and Down syndrome (DS), in comparison to controls with typical development (TD). While the present study focused primarily on comparisons between ASD-O and FXS-ASD, pragmatic difficulties are present in populations with neurodevelopmental disability other than ASD (e.g., FXS-O, DS, Williams syndrome), to varying degrees and with potentially unique profiles across conditions (30, 39–42). Thus, defining syndrome-specific pragmatic profiles can inform precision medicine across populations. Only males were included in this study due to the lower incidence of ASD in females (43), and given evidence that females with FXS are less affected than males overall, and specifically show lower incidence of co-morbid ASD (9, 31, 44) because of the protective nature of an additional X chromosome [e.g., (45)].

Multiple language contexts (standardized assessment, story narration, and semi-naturalistic conversation) were studied in order to examine how structural and social-communicative demands might reveal unique patterns of pragmatic challenges across groups. A longitudinal, developmental lens is particularly relevant when assessing pragmatic language, given that pragmatic demands increase dramatically with age in TD [e.g., (46)]. Longitudinal approaches are also especially relevant for neurodevelopmental disabilities; longitudinal research on disorders such as ASD-O (47, 48), DS (49), FXS (31), and attention deficit-hyperactivity disorder (50) demonstrate changes in both observable behaviors and neurobiology across the lifespan. It is also critical to understand the impact of communicative context on pragmatic skills to identify comprehensive, syndrome-specific profiles of strengths and weaknesses that might aid in the refinement of interventions. In ASD-O, for instance, evidence suggests that pragmatic impairments are most severely expressed in conversational contexts with high interpersonal demands, whereas less severe impariments are evident in structured communication contexts such as picturebook narration [e.g., (51)].

We further compared pragmatic profiles obtained through gold-standard, hand-coding methods assessing a range of key pragmatic skills, against computationally derived measures of pragmatic competence that have previously been applied in studies of narrative language in ASD (52–54). While conceptually and theoretically valid, and readily interpreted clinically, hand-coding methods are time intensive and can be difficult to obtain coding reliability. More efficient, objective computational measures for capturing pragmatic skills may therefore offer a valuable complement or eventual alternative to hand coding, that could be applied in large samples for use in both research and clinical practice (e.g., as measures of response to intervention). Vector semantic space models, such as latent semantic analysis (LSA), represent a promising method for characterizing pragmatic language in ASD. LSA produces a quantitative measure, ranging from-1 to 1, of how similar words, phrases or bodies of text are at a semantic level. Prior work has applied LSA to distinguish narratives of ASD from controls across two different samples and narrative tasks (52, 53). Importantly, both studies found that greater LSA scores (i.e., closer to 1) related to meaningful aspects of narrative, including complex syntax and narrative evaluation. Together, this work suggests that such an approach may hold utility for characterizing language and social communication in ASD. However, studies have yet to apply vector semantic methods to narrative from other neurodevelopmental disabilities, or to extend this method to less structured contexts.

In summary, the current study had three primary objectives: (1) to comprehensively characterize pragmatic language development over time across groups, with a focus on ASD-related pragmatic profiles that may emerge across idiopathic and syndromic ASD; (2) to examine the role of assessment context on pragmatic language profiles, and whether the impact of context differs between groups; and (3) to assess the utility of computational tools for measuring pragmatic language differences across groups and over time. We hypothesized that (1) all clinical groups would differ from controls and develop pragmatic skills more slowly over time, but boys with ASD-O and FXS-ASD would demonstrate specific areas of overlap and differences in pragmatic profiles that would persist over time; and (2) pragmatic differences in ASD-O and FXS-ASD would be more pronounced in the least structured pragmatic context, with context playing less of a role for boys with FXS-O and DS. Application of computational measures was more exploratory, with the goal of validating these novel measures against gold-standard hand coding and examining utility for distinguishing clinical group profiles and links to indices of pragmatic skill within groups. Overall, this study aimed to inform personalized medicine (assessment and intervention) and research approaches, and to potentially identify shared ASD-related pragmatic phenotypes (in ASD-O and FXS-ASD) that may suggest common genetic influences related to ASD. This study also aligns with a neuro-constructivist approach to language development, which acknowledges the interaction of genes, brain, cognition, and environment (55).

## 2. Materials and methods

### 2.1. Participants

Participants included 43 boys with ASD-O, 71 boys with FXS (57 with FXS-ASD; 14 with FXS-O), 22 boys with DS, and 24 boys with TD. Participants were part of a longitudinal study of pragmatic language development in which assessments were completed approximately once per year for up to three time points. Some children had fewer time points due to a “rolling enrollment” structure of the study where participants who enrolled later did not have time for multiple yearly visits before the study ended, as described previously (56). The number of participants receiving each assessment at each time point is summarized in Table 1. Boys with ASD-O, FXS, and DS were recruited from research registries, genetic clinics, parent support groups, and physician’s offices in the Eastern and Midwestern United States. Controls with TD were recruited locally from research registries, childcare centers, schools, and physicians’ offices.

**Table 1:** Number of participants by group with each assessment at each visit.

Visit	Group	CASL-PJ	Narrative	PRS-SA
1	ASD-O	37	22	35
	FXS-ASD	52	30	42
	FXS-O	15	10	14
	DS	21	18	21
	TD	23	15	22
2	ASD-O	20	––	4
	FXS-ASD	43	––	4
	FXS-O	11	––	1
	DS	20	––	––
	TD	18	––	1
3	ASD-O	15	9	15
	FXS-ASD	32	19	27
	FXS-O	5	4	5
	DS	14	11	14
	TD	11	7	11

Narrative data were not collected at the second visit. ASD-O, autism spectrum disorder only; FXS-ASD, fragile X syndrome with ASD; FXS-O, FXS only; DS, Down syndrome; TD, typical development; CASL-PJ, Comprehensive Assessment of Spoken Language-Pragmatic Judgment Subtest; PRS-SA, Pragmatic Rating Scale-School Age.

Inclusion criteria for all children included speaking English as their first and primary language, and using at least three-word phrases at the time of enrollment. Genetic testing confirming the full mutation of the *FMR1* gene was also necessary for boys with FXS-ASD and FXS-O. The Autism Diagnostic Observation [ADOS (57)] was administered to confirm diagnosis in boys with ASD-O and determine ASD status in boys with FXS. Boys with FXS were classified as FXS-ASD if their average severity score (across longitudinal assessments) was consistent with an ASD classification as defined by updated ADOS algorithms (58, 59). Average severity scores were used to ensure consistency in classification over time and to reflect a best estimate classification based on the most information available.

Exclusion criteria for all children included failure to pass a standard hearing screening. Boys with DS and TD could not meet ASD criteria on the ADOS at any point over the course of the longitudinal study. Boys with TD could also have no history of language or other developmental delays.

Although we aimed to match groups on nonverbal mental age, this proved difficult given the rarity of some of our groups. Therefore, analyses included nonverbal mental age as a covariate. Nonverbal mental age was assessed using the Leiter International Performance Scale-Revised (60). Table 2 summarizes chronological age and mental age across groups and time points. Institutional review boards approved all procedures.

**Table 2:** Chronological age and mental age at each visit.

	Group	Chronological age (years) *M* (SD) range	Nonverbal mental age (years)^1^ *M* (SD) range
Visit 1	ASD-O	8.27 (2.90)^a^ 3.24–13.27	6.11 (1.8)^a^ 2.33–10.50
	FXS-ASD	10.56 (2.47)^b^ 6.58–15.07	5.00 (0.56)^b^ 3.50–6.25
	FXS-O	9.34 (3.24)^a,b^ 5.59–14.98	5.64 (1.42)^a,b^ 3.67–9.17
	DS	10.94 (2.07)^b^ 6.81–14.86	5.33 (0.81)^b^ 4.33–8.25
	TD	4.74 (1.10)^c^ 3.15–7.07	5.23 (1.21)^b^ 3.58–7.67
Visit 2	ASD-O	10.31 (2.20)^a^ 6.41–13.92	6.34 (1.57)^a^ 4.42–10.25
	FXS-ASD	12.02 (2.53)^b^ 7.95–16.75	5.07 (0.58)^b^ 3.25–6.58
	FXS-O	11.47 (3.56)^a,b^ 7.50–16.40	5.72 (1.29)^a,b^ 4.50–9.17
	DS	12.39 (2.03)^b^ 7.93–16.09	5.66 (1.09)^a,b^ 3.08–8.25
	TD	6.41 (1.54)^c^ 4.60–10.33	6.80 (1.65)^a,c^ 5.58–11.67
Visit 3	ASD-O	11.69 (2.34)^a^ 7.54–15.77	6.94 (1.71)^a^ 4.42–10.25
	FXS-ASD	13.10 (2.55)^a,b^ 9.10–17.90	5.15 (0.55)^b^ 4.42–6.67
	FXS-O	11.64 (2.87)^a,b^ 8.73–16.38	5.11 (0.75)^b^ 4.00–6.00
	DS	14.14 (2.51)^b^ 9.63–17.93	5.99 (1.33)^a,b^ 4.58–9.58
	TD	7.73 (1.70)^c^ 6.15–11.55	8.49 (3.11)^c^ 6.00–17.08

1 Leiter International Performance Scale-Revised.

ASD-O, autism spectrum disorder only; FXS-ASD, fragile X syndrome with ASD; FXS-O, FXS only; DS, Down syndrome; TD, typical development. Per visit, different superscripts within a column indicate significant differences at *p* < 0.05. If groups share the same letter, differences were not significant.

### 2.2. Pragmatic language assessment contexts (non-computational analyses)

#### 2.2.1. Standardized measure

The Comprehensive Assessment of Spoken Language-Pragmatic Judgment Subtest [CASL-PJ (61)] measured how participants would respond to certain social situations (e.g., “how would you greet an unfamiliar adult?”). Age equivalencies were used as the outcome variable in all analyses.

#### 2.2.2. Narrative

Participants viewed the short, silent cartoon, *Pingu’s Parents Go to a Concert* (62), which is about a penguin “child” and siblings who engage in various behaviors while their “parents” are gone (e.g., overfilling a bathtub, jumping on the bed). Participants first viewed the video on a laptop with standardized examiner prompts to emphasize key plot points (e.g., “look, the parents are leaving”). They were then shown the video again and asked to tell the examiner the story while viewing the video. Narratives were transcribed with Systematic Analysis of Language Transcripts [SALT; (63)] software, and coded for key elements of narrative using a coding scheme adapted from prior work (51, 64–69). Narratives were transcribed by research assistants who were first trained to 80% word and utterance segmentation reliability. A second, independent research assistant transcribed 10% of files from each diagnostic group to assess transcription reliability; mean word agreement was 87.5% and mean utterance segmentation was 83.3%.

Coding produced scores for (1) length (total propositions, defined as a verb and its arguments), (2) story grammar (e.g., key story elements such as introduction, plot points and conclusion), (3) character relationships, (4) audience engagement (i.e., devices used to get and maintain listener attention), and (5) mention of character thoughts and emotions, and causal connections. A narrative summary score was also calculated for use in analyses comparing cross-context effects (summed proportions of each narrative element to total utterances). Intercoder reliability was assessed for 10% of files; all primary narrative outcome variables had an intraclass correlation coefficient (ICC; 3.2) greater than 0.9, signifying “excellent agreement,” apart from off-topic (utterances unrelated to the story, such as asking about goldfish or some other unrelated topic). with an ICC of 0.62, representing “good” agreement (70).

#### 2.2.3. Semi-naturalistic conversation

To assess pragmatic language during semi-naturalistic conversational interactions, videos of the child and examiner interacting during the ADOS were rated using the Pragmatic Rating Scale-School Age [PRS-SA; (71)]. The PRS-SA is a clinical-behavioral rating system of 34 operationally defined features of pragmatic language assessing a range of pragmatic language features. Each item is rated on a three-point scale (0, absent; 1, mild impairment; or 2, impairment present); items are then totaled to provide an overall sum of pragmatic violations (PRS-SA Total). Additionally, the PRS-SA includes theoretically defined subscales of skills: *theory of mind* (e.g., failure to provide background information, providing too much detail), *discourse management* (e.g., limited topic initiation and elaboration, reduced acknowledgment of conversational partner), *speech and language behaviors impacting pragmatics* (e.g., overly formal speech, repetitive speech), *suprasegmentals* (e.g., atypical prosodic features of speech such as rate, volume and fluency), and *nonverbal communication* (e.g., eye contact, facial expressions).

PRS-SA ratings were completed by research team members who were either trained by the test developer, or who maintained 80% reliability with a team member who was trained by the test developer. Fifty-two total files (approximately 20% of each group) were assessed for reliability. ICCs indicated overall reliability as 0.86 (0.71 for ASD, 0.80 for FXS, 0.84 for DS and 0.84 for TD), signifying “good-excellent agreement” (70).

### 2.3. Computational analysis of narrative and semi-naturalistic conversation

#### 2.3.1. Vector semantics: comparison to gold standard

Word2Vec, a vector semantic model, was applied to quantify narrative quality, measured through the semantic similarity of narratives and conversation to “gold standards” derived from the TD group. This automated measure produces a quantitative measure of similarity, ranging from –1 to 1, for each text relative to the respective gold standard. Vector semantic models are first “trained” on large corpora of text [in this case, Google news embeddings (72)] to “learn” the frequency of co-occurrence of words in semantic space. Subsequently, each participant’s transcript is processed through the model to create a 400-dimension vector space representation of all the words included, which is then reduced by summing and normalizing the vector. The semantic “distance” of the sum of all the word vectors from a given transcript to the gold standard vector is then calculated (i.e., the cosine between vectors), resulting in a single, quantitative measure of semantic similarity ranging from –1 to 1, with 1 being the most similar. The scripts used to process these transcripts were developed by two of the authors (Bicknell and Goodkind) and are available on an open-source repository.

For the narratives and semi-naturalistic conversations, gold standards were selected from the TD control group based on representativeness of pragmatic competence for that context (e.g., inclusion of evaluation and story elements in narrative; lack of pragmatic violations in conversation). Gold standards were excluded from all other analyses. For all Word2Vec analyses, partially or completely unintelligible utterances, as well as mazes (i.e., repetitions and reformulations within an utterance) were excluded from analyses to maximize intelligibility and avoid inflation of semantic content from reformulation as possible confounds.

#### 2.3.2. Vector semantics: exchange similarity

For the semi-naturalistic conversational context, vector semantics were also applied to compute semantic similarity (−1 to 1) between the child and examiner utterances across conversational “exchanges,” to capture pragmatic coherence in conversation. An “exchange” was defined as two conversational turns (i.e., the child’s turn and the examiner’s turn that followed it, or vice versa, where a turn could consistent of one or more utterances), similar to the definition of conversation used in the ADOS (57). The mean semantic similarity score across all exchanges in a transcript was computed for each participant.

### 2.4. Analysis plan

Analyses were conducted to (1) examine group differences and change over time within each context (standardized assessment, narrative, semi-naturalistic conversation), (2) compare each group’s performance across contexts at visit one, and (3) explore the utility of computational analysis of pragmatic competence during narrative and semi-naturalistic conversation by comparing computational and gold-standard hand-coding measures. These analyses are described in greater detail below.

#### 2.4.1. Group differences and change over time within contexts

##### 2.4.1.1. Standardized assessment (CASL-PJ)

A hierarchical linear model (HLM) was conducted, with age as a marker of time nested within participant, to assess the main effect of group, the main effect of age, and interaction between age and group (i.e., whether rates of development differed across groups), covarying for mental age. All results are reported as fixed effects; random intercepts of age were also included in the model. Chronological age and mental age were grand mean centered to reduce collinearity.

##### 2.4.1.2. Narrative

Primary analyses of narrative occurred at visit one due to lower sample sizes at subsequent visits. To examine group differences, Poisson analyses for primary outcome variables at visit one were conducted, including mental age as a predictor and offset by the log of propositions (an index of length). In cases where examination of residual plots indicated skew, these analyses were followed up by a more conservative approach of binary logistic regressions with group as a predictor and mental age as a covariate to determine whether group status predicted an amount of a narrative element below or above the overall mean. To assess change over time, within-group Wilcoxon Ranked Sum Tests assessed changes in proportions of narrative features across time points within each group.

##### 2.4.1.3. Semi-naturalistic conversation (PRS-SA)

An HLM was also conducted for the PRS-SA total score, as well as for each theoretically derived subscale, with age as a marker of time nested within participant, to assess the main effect of group, the main effect of age, and interaction between age and group, covarying for mental age. All results are reported as fixed effects; random intercepts and random slopes of age were attempted but could not be validly fit to the model. To reduce collinearity, chronological age and mental age were grand mean centered. Overall analyses were followed by planned pairwise comparisons.

#### 2.4.2. Context analysis across groups at visit one

The role of assessment context was examined by calculating Z-scores comparing the means of each individual to the mean of the TD group for each context at visit one: standardized assessment (CASL-PJ age equivalent), narrative ability (narrative summary score), and semi-naturalistic conversation (total number of violations on the PRS-SA). Repeated measures then compared group changes in Z-scores across the contexts at visit one, controlling for mental age.

#### 2.4.3. Utility of computational analysis in characterizing pragmatic competence across groups

Group comparisons for each computational outcome variable were conducted using non-parametric Kruskal-Wallis one-way analysis of covariance, followed by planned comparisons. To assess change over time for computational analyses, within-group Wilcoxon Ranked Sum Tests were conducted from the first to second visit to determine whether computational measures were sensitive to change over time. Finally, Pearson bi-variate correlations assessed relationships between primary computational outcome measures and narrative and semi-naturalistic conversation coding variables.

## 3. Results

### 3.1. Group differences and change over time within contexts

#### 3.1.1. Standardized assessment (CASL-PJ)

**Figure 1:**
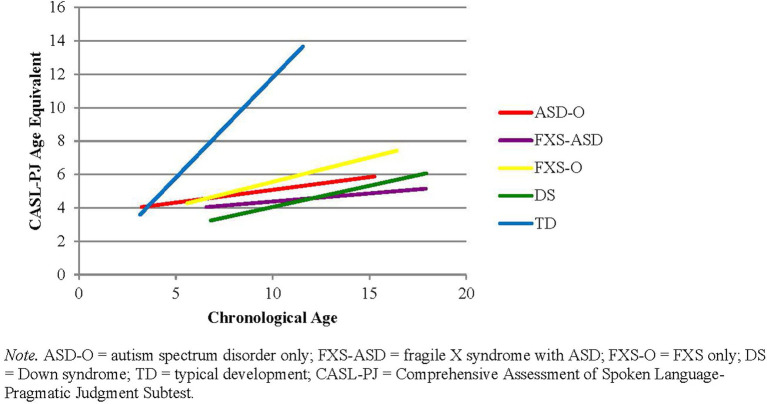
Changes in CASL-PJ age equivalence with age.

Results of the HLM revealed a significant main effect of group (*F* = 27.77, *p* < 0.001), age (*F* = 70.47, *p* < 0.001), and group by age interaction for the CASL-PJ age equivalent score (*F* = 11.32, *p* < 0.001). Boys with ASD-O, FXS-ASD, and DS performed significantly more poorly than boys with FXS-O (*p* values <0.05), and all groups performed lower than the TD group (p values <0.001). Whereas the TD group showed growth in age equivalence with chronological age (slope = 0.77), and the FXS-O group showed moderate growth (slope = 0.30), other clinical groups were relatively stable (ASD-O = 0.14, FXS-ASD = 0.12, DS = 0.15). Figure 1 demonstrates rates of change in CASL-PJ scores for the different groups.

#### 3.1.2. Narrative

**Figure 2:**
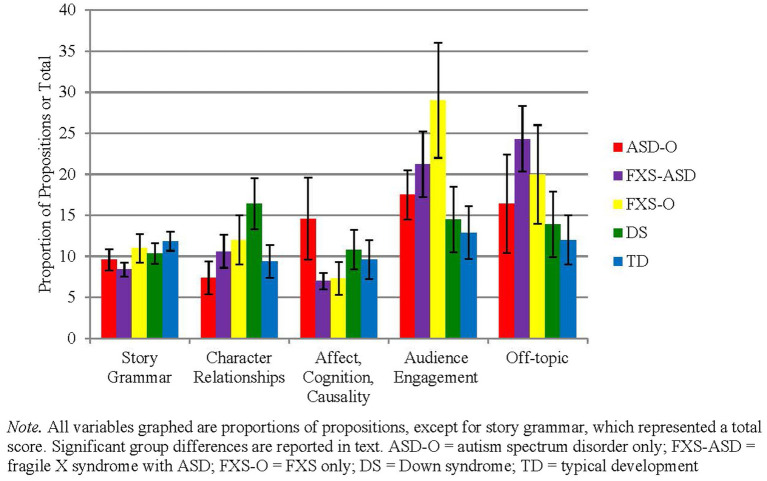
Profiles of narrative features at visit one.

There was an overall main effect of group on inclusion of narrative elements (Wald = 11.2, *p* = 0.025). The ASD-O group did not differ from the FXS-ASD group in total elements (*p* = 0.50) but included significantly fewer elements than the TD and DS groups (ps <0.05). There was also a significant effect of group on inclusion of character relationships (Wald = 21.32, *p* < 0.001), in that boys with DS included significantly more character relationships than the ASD-O, FXS-ASD and TD groups, and boys with ASD-O included significantly fewer character relationships than boys with FXS-ASD and FXS-O (pairwise ps < 0.03). Boys with FXS-O used the greatest amount of strategies to engage the listener overall, followed by boys with FXS-ASD, who used significantly more than all other groups (Wald = 54.01, *p* < 0.001; binomial logistic regression: overall Wald for group = 9.12, *p* = 0.06; pairwise comparisons ps < 0.05 for FXS-O group only). There were no group differences in total inclusion of character thoughts and feelings (ps > 0.1). Figure 2 summarizes patterns of group performance on narrative variables at the initial visit.

Overall, patterns of narrative device use were largely stable within groups across time points, although both males with FXS-ASD (*Z* = −2.0, *p* = 0.047) and TD (*Z* = −2.4, p = 0.02) increased their inclusion of character relationships across visits. Males with TD also increased their inclusion of story elements (*Z* = 2.1, *p* = 0.03).

#### 3.1.3. Semi-naturalistic conversation (PRS-SA)

**Figure 3:**
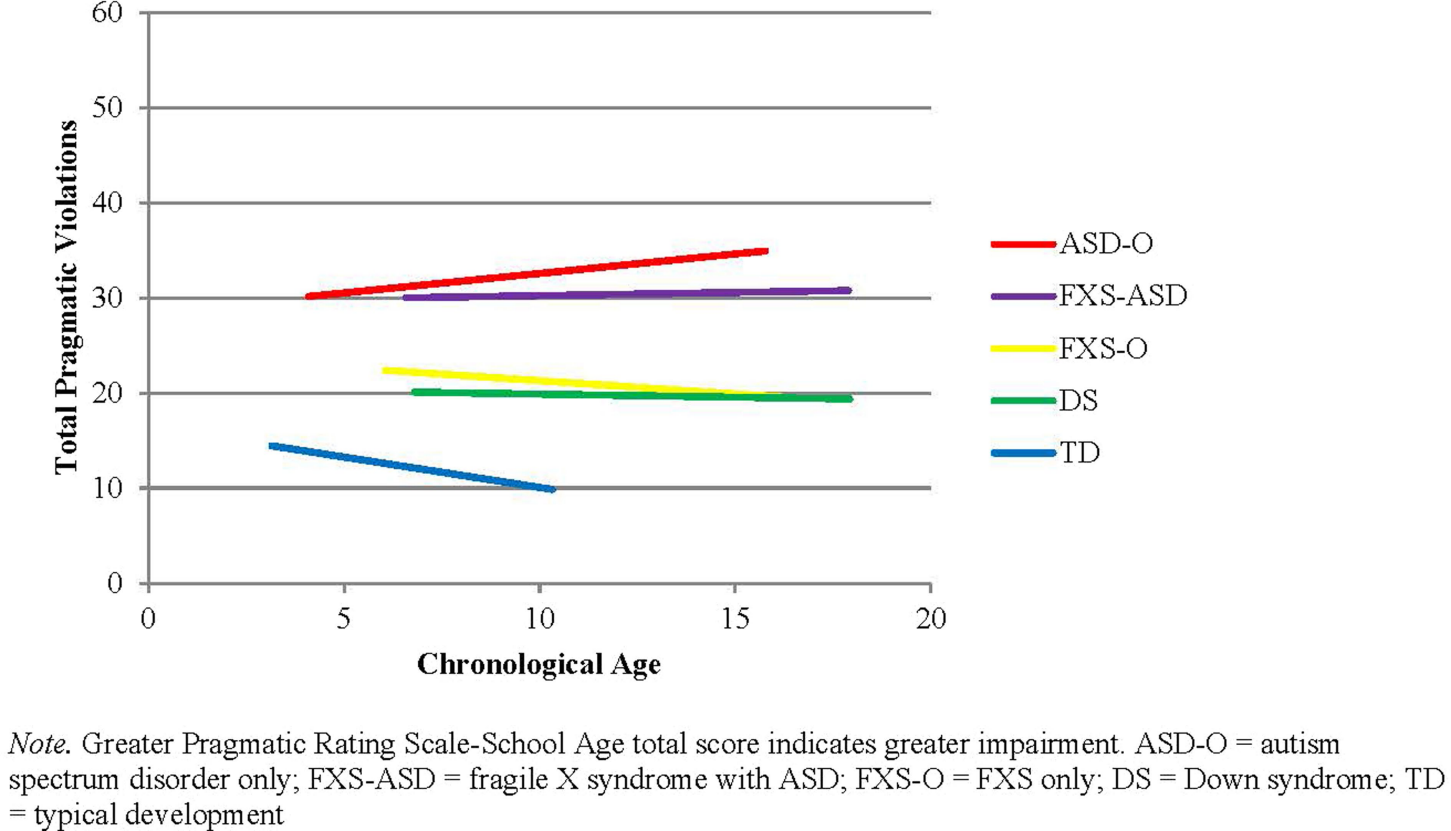
Overall changes in pragmatic violations with age.

Hierarchical linear models assessed changes in PRS-SA total with age, as well as the theoretically derived subscales of the PRS-SA. There was a main effect of group for all outcome variables, but no main effects of age or group * age interactions (Fs < 1, ps > 0.30). For total pragmatic impairment, boys with ASD-O showed the most significant pragmatic impairment, followed by boys with FXS-ASD, who showed significantly more pragmatic violations than boys with FXS-O, DS, and TD (pairwise comparisons: ps < 0.05). Trajectories of total pragmatic violations are summarized in Figure 3.

Subscale findings are shown in Table 3. For the discourse management subscale, there was an overall main effect of group (*F* = 14.03, *p* < 0.001). Pairwise comparisons revealed that the ASD-O and FXS-ASD groups did not significantly differ (*p* = 0.096), and both groups showed greater impairment than boys with FXS-O and DS (ps < 0.001); the ASD-O group also showed greater impairment than boys with TD (*p* = 0.011). Boys with ASD-O and FXS-ASD similarly did not differ (*p* = 0.71) and showed significantly greater impairment in suprasegmental features of speech than all other groups; boys with FXS-O also showed greater impairment than boys with TD (*p* = 0.031; group main effect *F* = 6.61, *p* < 0.001). For nonverbal aspects of pragmatics (*F* = 25.99, *p* <. 001), boys with ASD-O and FXS-ASD did not significantly differ (*p* = 0.07) and showed greater impairment than all other groups (ps < 0.05). The main effect of group for the theory of mind subscale (*F* = 4.73, *p* = 0.001) was driven by boys with ASD-O showing significantly greater impairment on this subscale than boys with FXS-ASD, FXS-O, and DS (pairwise ps < 0.01). Finally, all clinical groups showed greater impairment than boys with TD in speech and language behaviors contributing to pragmatic language (*F* = 3.67, *p* < 0.001; pairwise ps < 0.01).

**Table 3:** Estimated marginal means from HLM models for PRS-SA subscales.

	Discourse management	Suprasegmental features of speech	Nonverbal aspects	Theory of mind	Speech and language behaviors contributing to pragmatics
Group	Mean (SE)	Mean (SE)	Mean (SE)	Mean (SE)	Mean (SE)
ASD-O	10.13 (0.57)	5.75 (0.36)	8.03 (0.35)	5.54 (0.41)	3.93 (0.23)
FXS-ASD	8.78 (0.50)	5.55 (0.32)	7.11 (0.31)	3.87 (0.36)	4.44 (0.20)
FXS-O	4.96 (0.81)	4.22 (0.52)	4.37 (0.51)	3.62 (0.59)	3.80 (0.33)
DS	4.69 (0.76)	3.89 (0.50)	3.28 (0.48)	2.92 (0.56)	3.89 (0.31)
TD	5.24 (1.97)	1.06 (1.34)	2.57 (1.31)	3.14 (1.52)	1.24 (0.85)

Model included chronological age and mental age, mean centered. ASD-O, autism spectrum disorder only; FXS-ASD, fragile X syndrome with ASD; FXS-O, FXS only; DS, Down syndrome; TD, typical development; PRS-SA, Pragmatic Rating Scale-School Age.

### 3.2. Context analysis across groups

**Figure 4:**
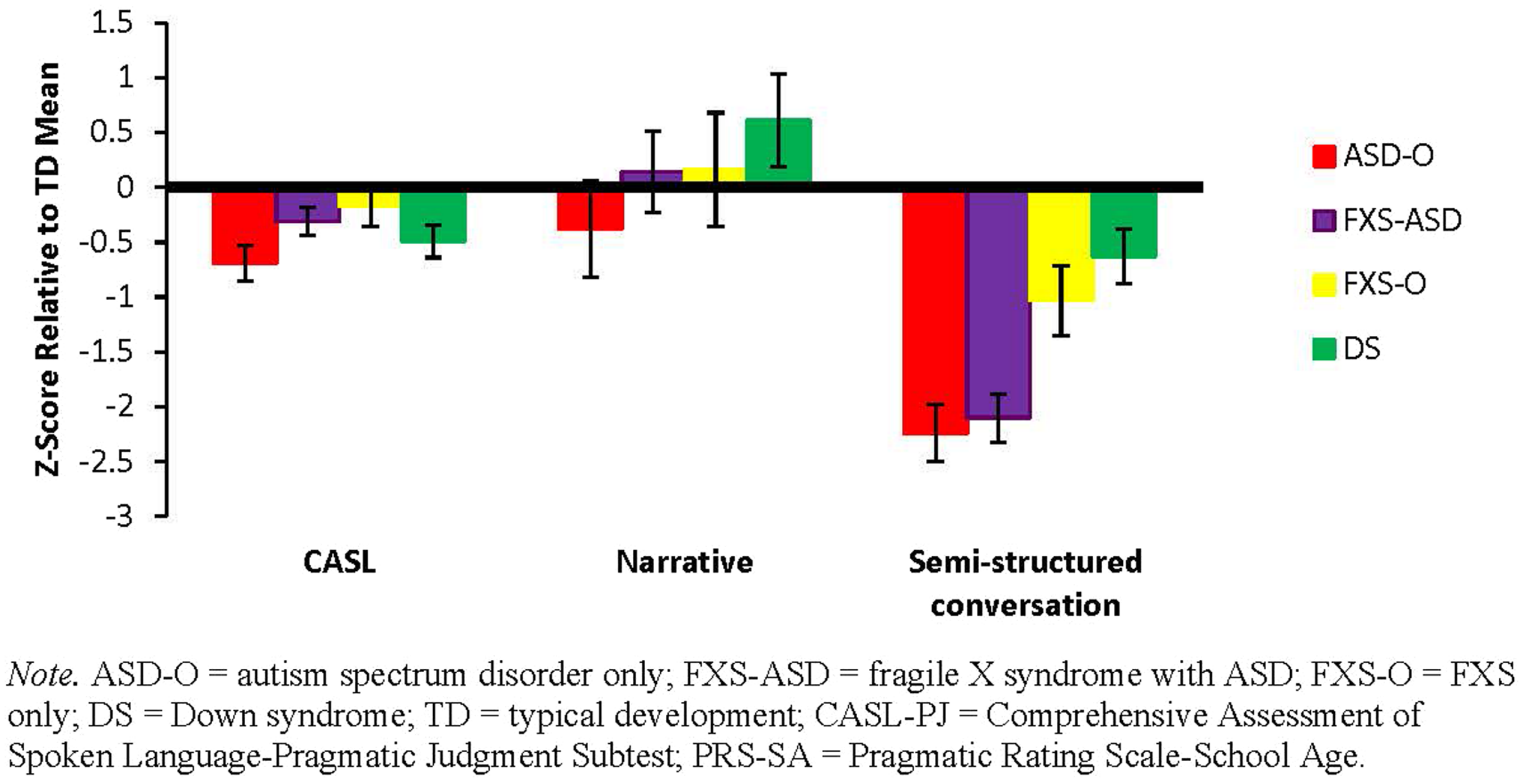
Context analysis across groups at visit one (Z-scores comparing means to TD group mean): males with ASD-O and FXS-ASD demonstrated a steeper decline between the standardized and narrative tasks relative to the semi-naturalistic conversation task.

There was a significant group by context interaction (*F* = 4.69, *p* < 0.001) and main effect of group (*F* = 4.25, *p* = 0.01) in patterns of performance across contexts. As summarized in Figure 4, boys with ASD-O and FXS-ASD demonstrated a steeper decline between the standardized and narrative tasks relative to the semi-naturalistic conversation task and showed greater overall deviation from the TD group relative to boys with DS.

### 3.3. Utility of computational analysis in characterizing pragmatic competence

For narrative, no group differences were observed in similarity to the gold standard at visit one (*H* = 6.30, *p* = 0.18). Within-group repeated measures revealed no significant changes over time in semantic similarity to a gold standard (Zs < 1.42, ps > 0.16). Across groups, higher semantic similarity to the gold standard was associated with increased total story elements in the ASD-O, FXS-ASD, DS and TD groups (rs > 0.59, ps < 0.05), and with nonsignificant, but medium-large effect, in the FXS-O group (*r* = 0.39). Additionally, greater semantic similarity was significantly related to reduced use of devices to engage the listener in the FXS-ASD group only (*r* = −0.51, *p* < 0.05), whereas use of audience engagement devices was correlated with medium effect size within the males with FXS-O (*r* = 0.39).

For semi-naturalistic conversations, comparisons to the TD gold standard distinguished all clinical groups from controls with TD at the initial visit (overall model: *H* = 20.61, *p* < 0.001, pairwise comparisons ps < 0.05). All clinical groups also demonstrated a lower mean exchange similarity with examiners relative to the TD group (*H* = 20.31, *p* < 0.001; pairwise comparisons ps < 0.05). There were no significant within-group changes across time points for similarity to the gold standard or average interchange similarity (Ws < 1.61, ps > 0.1). Exchange similarity was associated with reduced pragmatic violations related to reciprocity during the semi-naturalistic conversation in both the ASD-O and FXS-ASD groups (absolute value of *r* > 0.3, ps < 0.05). Reduced semantic similarity to the gold standard was also correlated with greater total pragmatic violations in the FXS-O and DS groups (rs < 0.45, ps < 0.05).

## 4. Discussion

This study aimed to perform comprehensive characterization of pragmatic skills across multiple contexts, and examine change over time across multiple neurodevelopmental conditions in which pragmatics skills are variably impacted, with a focus on ASD-specific pragmatic profiles that may be present in both idiopathic and syndromic ASD. To our knowledge, this is the first longitudinal examination of pragmatic language, across multiple contexts, in these groups. Understanding overlap and differences in pragmatic profiles across conditions, and specifically in two different manifestations of ASD (idiopathic and syndromic), has important implications for precision medicine approaches to tailoring clinical interventions based on refined clinical symptom profiles, and where distinct etiologic factors may be at play. Findings confirmed the hypothesis that the ASD groups (i.e., ASD-O and FXS-ASD) would show similar (but not identical) pragmatic profiles across all three contexts, with more difficulties in the least structured context (semi-naturalistic conversation), consistent with a pragmatic profile previously documented in idiopathic ASD [e.g., (51)]. Patterns of group differences were more nuanced for boys with FXS-O and DS, with structure having less of an impact on pragmatic skills. Overall, clinical groups demonstrated minimal changes in pragmatic skills with age. Results suggest that novel computational language measurement of pragmatic abilities may hold some utility, particularly for conversation, but are not currently as precise as traditional hand-coding measures for detecting differences between clinical groups. We elaborate on these findings below.

### 4.1. Group profiles across contexts varying in structure

#### 4.1.1. Standardized measure

The Pragmatic Judgement subtest of the CASL represented the most structured assessment of pragmatic skills in the present study. On this measure, all clinical groups performed more poorly than the TD control group, and the boys with ASD-O, FXS-ASD, and DS also performed more poorly than boys with FXS-O. Additionally, the ASD-O, FXS-ASD, and DS groups showed little change over time. These findings are generally consistent with those of Martin et al. (73), who also found that boys with TD scored higher and showed more change over time than boys with FXS and DS on the same measure (boys with ASD-O did not participate); in this study, as in results presented here, boys with FXS-O also outperformed boys with FXS-ASD in pragmatic judgment. Together, these results indicate that pragmatic language deficits exist across all clinical groups as assessed by a standardized measure, and provide evidence for the impact of ASD status on pragmatic deficits in FXS. Note, however, that the DS group performed similarly to both ASD groups in the present study, suggesting that results of standardized pragmatic assessments may not always distinguish ASD from other forms of neurodevelopmental disability.

#### 4.1.2. Narrative

The narrative task represented an intermediate degree of structure between the standardized assessment and the semi-naturalistic conversation. The two ASD groups did not differ from each other in total story elements, and both groups used fewer story elements overall than boys with DS and TD. However, boys with ASD-O included significantly fewer character relationships than boys with FXS-ASD (and FXS-O), highlighting both similarities and differences in idiopathic ASD and syndromic ASD associated with FXS. Previous research on narrative in FXS has been limited, and with mixed findings (65, 66, 74, 75). In the current study, while ASD status impacted narrative quality in FXS to the extent that, consistent with Estigarribia et al. (65), boys with FXS-ASD used fewer story elements than boys with FXS-O, a notable feature of narratives of both FXS groups was their increased use of audience engagement devices. Although attempts to engage the audience in a story can contribute to successful narration (69, 76, 77), excessive attempts to do so may detract from narrative quality (78). Consistent with prior work suggesting that narrative may represent an area of relative strength for males with DS (74, 75, 79, 80), boys with DS included the greatest number of character relationships in their narratives. Of note, although most studies suggest that narrative is a relative strength in DS, Channell et al. (81) did report that young individuals with DS included fewer episodic elements than controls matched on nonverbal cognitive ability. However, these group differences were explained by mean length of utterance, suggesting that this finding was more reflective of grammatical than pragmatic deficits.

#### 4.1.3. Semi-naturalistic conversation

Semi-naturalistic conversation that occurred during the ADOS was the least structured of the three contexts examined. Assessment of overall pragmatic deficits in this context revealed a graded pattern of results, where boys with ASD-O demonstrated the greatest difficulty, followed by boys with FXS-ASD, who showed greater impairments than boys with FXS-O, DS, and TD. Patterns of group differences on theoretically derived subscales revealed areas of overlap and divergence in boys with ASD-O and FXS-ASD, consistent with previous studies (29, 30, 38). Both groups showed deficits in discourse management, suprasegmental features, and nonverbal aspects of pragmatics. However, boys with ASD-O showed more difficulty than boys with FXS-ASD on items thought to be associated with theory of mind (e.g., failure to provide necessary background information, recognizing communication breakdowns). Links between pragmatic impairment and theory of mind in idiopathic ASD are well-documented (32, 51, 64, 82–86), and may represent an important distinction between idiopathic and syndromic ASD associated with FXS that should be examined further in future studies.

### 4.2. Change over time

This study was novel in its inclusion of multiple time points during the school-age years for individuals with neurodevelopmental conditions, a time when pragmatic language becomes increasingly complex (and relevant to social relationships) in TD (87). The TD group—and, to a lesser extent, the FXS-O group—showed growth over time on the standardized measure, whereas the other groups did not. This improvement is expected for the TD group given the use of age equivalents in analysis, and is a promising finding for males with FXS-O. However, the lack of substantial growth in the ASD-O, FXS-ASD, and DS groups suggests that deficits do not improve considerably over time and, moreover, that the divergence with TD widens with age. The TD group also included more story elements in their narratives over time, with no such growth noted in the clinical groups. A more surprising finding was that both the TD group *and* the FXS-ASD group included more character relationships in their narratives over time. This is consistent, however, with the FXS-ASD group including more character relationships than males with ASD-O overall, offering further support for this particular aspect of narrative representing an important difference between idiopathic ASD and FXS-ASD. None of the groups, including the TD group, showed significant change with age in pragmatic skills during semi-naturalistic conversation. These findings likely reflect insufficient measurement sensitivity for detecting change over time, rather than true arrestment in the development of conversational pragmatic skills.

### 4.3. The role of context

Examining pragmatic skills across multiple contexts revealed another key area of overlap between boys with ASD-O and FXS-ASD—the marked increase in difficulties during a less structured versus more structured context. Prior work has suggested that males with ASD-O benefit from increased structure in discourse contexts (51, 88–90). Current results confirm these findings and extend them to males with FXS who also meet criteria for ASD, suggesting that impairments in both groups are likely to be most prominent in less structured contexts. In contrast, males with FXS-O and DS showed less variation across contexts, suggesting that the degree to which structure supports pragmatic competence may be unique to the ASD phenotype regardless of FXS status, and should be a key component of pragmatic interventions for both forms of ASD.

### 4.4. Utility of computational analysis in characterizing pragmatic competence

Computational approaches for characterizing pragmatic language are attractive as an objective efficient tool that could complement (and potentially replace) time and labor intensive manual coding methods. However, results from analysis of vector semantic similarity methods applied to narrative and semi-naturalistic conversational samples suggest that these methods, at least in their current form, are still a relatively blunt tool not yet capable of capturing nuanced patterns of differences evident in hand coding results. Indeed, semantic similarity analyses distinguished all clinical groups from controls with TD in the semi-naturalistic conversational context only. All clinical groups also demonstrated a lower mean exchange similarity with examiners relative to the TD group in conversation. However, these measures did not show differences between clinical groups, despite differences detected using hand coding, and a robust body of literature suggesting differences in pragmatic language between groups [e.g., (29, 30, 37, 73)]. Perhaps more encouraging was that, for conversation, variation in exchange similarity was associated with reduced pragmatic violations related to reciprocity in both the ASD-O and FXS-ASD groups. Reduced semantic similarity was also associated with greater overall pragmatic violations in the FXS-O and DS groups, suggesting that computational analytical methods may hold promise for pragmatic characterization in these groups as well.

While group differences were not detected for the narrative context using computational analytical methods, semantic similarity was related to inclusion of more story elements across groups, and reduced use of devices to engage the listener in the FXS-ASD group (with a similar but non-significant relationship found for the FXS-O group), suggesting that this metric may be sensitive to meaningful aspects of narration. Change over time was not detected for either context using computational methods. Overall, results suggest that vector semantic analyses may hold some utility as an index of pragmatic language features, particularly with further refinement. However, this approach appears less sensitive to group-specific pragmatic differences that are critical to capture in biological and clinical intervention studies.

### 4.5. Potential clinical and biological implications

Results of this study may have several important implications for clinical assessment and intervention studies focused on pragmatic language across neurodevelopmental conditions. First, these findings highlight the importance of multi-method assessment, particularly in ASD-O and FXS-ASD. As predicted, the two ASD groups showed the most difficulties across contexts. Even so, and perhaps more interestingly, results suggest that important pragmatic differences may be masked by structured clinical assessments in these groups, underscoring the importance of conversational interaction in particular for assessing pragmatics. The conversational task examined in this study also more closely resembles the communication contexts most prevalent in daily life, which can pose specific pragmatic challenges that impact social interaction. These results suggest that clinical assessment and intervention should target conversational skills specifically, for idiopathic and syndromic ASD associated with FXS.

Results also revealed somewhat unique pragmatic profiles across the different clinical groups, highlighting unique clinical needs that may be targeted with a personalized medicine approach. For example, boys with FXS (regardless of ASD status) may require specific support in the regulation of pragmatic behaviors that detract from narrative, such as excessive seeking of attention, that was not characteristic of either the ASD-O or DS group. Further, while they showed more similarities than differences, boys with ASD-O and FXS-ASD differed in regard to including character relationships in their narratives and pragmatic features in conversation thought to be related to theory of mind. Of course, it is critical that ASD status be assessed in FXS, as boys with FXS-ASD demonstrated greater deficits than boys with FXS-O across contexts. Lack of growth for the clinical groups on the standardized measure of pragmatics (except for FXS-O) and narrative (except for FXS-ASD in a single aspect of narrative), suggest that pragmatic impairment generally persists over time and continues to be an area of need throughout the school years. Finally, as discussed previously, results of computational analyses suggest that while this method shows some relation to more time-intensive assessment methods, computational analyses do not currently offer the same utility as more traditional methods.

Overlapping pragmatic signatures in ASD-O and FXS-ASD groups can also inform understanding of potential influence of the *FMR1* gene on pragmatic language, a critical clinical dimension of ASD. The two groups of boys with ASD both showed more difficulty with less structure, as well as similar deficits in inclusion of story elements during narration and pragmatic language during conversation with the forementioned exceptions. Such specific areas of common difficulty may help to identify aspects of pragmatic language that can serve as fruitful targets of investigation of pathways from *FMR1* to ASD-related phenotypes.

### 4.6. Limitations and future directions

There are several limitations of the current study that should inform future research. First, it is critical for future studies to examine whether the profiles identified here extend to females across these different neurodevelopmental conditions, given sex differences observed across clinical groups in previous studies (15, 29, 56, 91, 92). Sex-specific patterns are well documented in FXS, owing to females’ second, unaffected X chromosome with normal *FMR1-*related protein expression and function. It is increasingly apparent that sex differences are evident in ASD as well, and that differences in pragmatics may be particularly relevant to understanding female-specific diagnostic profiles. Little work has examined such questions in DS, and establishing pragmatic differences in males and females will also be an important question to explore in this condition.

Additional longitudinal studies with larger samples, and following participants into adolescence and young adulthood would permit more robust characterization of pragmatic language growth trajectories and outcomes. Examining pragmatics in additional, naturalistic contexts would also be informative. For example, peer interactions would almost certainly provide less structure than an interaction with a trained examiner, with potential to reveal even more clinically relevant pragmatic differences for ASD groups than were revealed through the examiner-based measures studied here. Further, while we controlled for mental age in our analyses, other potentially related environmental variables such as ongoing interventions, opportunities for socialization, and the home environment along with other skills and abilities, such as structural language (e.g., vocabulary, syntax), theory of mind, and executive function, should be considered as potential contributors to pragmatic language difficulties. Finally, computational analysis with additional variables, larger samples, and making use of new computational developments should be conducted in future studies to further explore the utility of this approach for capturing pragmatic deficits in natural language samples.

### 4.7. Conclusion

In summary, this longitudinal study of pragmatics across neurodevelopmental conditions revealed potentially unique pragmatic language profiles in several groups, with important overlapping features also evident. Pragmatic language was most significantly impacted among males with ASD-O and FXS-ASD across all three contexts, with more difficulties in the least structured context (conversation). ASD-O and FXS-ASD profiles also differed, however, for certain aspects of narration and conversation. Boys with FXS-O and DS, the two groups with intellectual disability but not ASD, were more similar to each other than to either of the ASD groups, with context having less of an impact in these two groups. FXS-O and DS profiles did differ, however, with the DS group showing more evidence of a relative strength in narration. Computational language measurement tools were not as sensitive as traditional methods at capturing distinctive profiles across clinical groups. Better understanding of characteristic pragmatic language profiles has important implications for the development of precisely tailored assessment and intervention approaches for different forms of neurodevelopmental disability in general and different forms of ASD in particular.

## Data availability statement

The raw data supporting the conclusions of this article will be made available by the authors, without undue reservation.

## Ethics statement

The studies involving human participants were reviewed and approved by Institutional Review Boards at Northwestern University and the University of North Carolina at Chapel Hill. Written informed consent to participate in this study was provided by the participants’ legal guardian/next of kin.

## Author contributions

MLe, GM, and MLo contributed to conception and design of the study. MLe and NM completed data coding. KB and AG developed computational models and scripts used for computational analyses. MLe performed the statistical analysis and wrote the first draft of the manuscript with feedback from GM and MLo. GM revised the manuscript and prepared it for publication. MLo provided valuable feedback at all stages of manuscript preparation. All authors contributed to the article and approved the submitted version.

## Funding

This work was supported by grants from the National Institute of Child Health and Human Development (R01HD38819 and R01HD044935), the National Institute on Deafness and Other Communication Disorders (R01DC010191), and the National Institute of Mental Health (R01MH091131). All funding was used toward data collection and analysis and salary support.

## Conflict of interest

KB was employed by the company Duolingo.

The remaining authors declare that the research was conducted in the absence of any commercial or financial relationships that could be construed as a potential conflict of interest.

## Publisher’s note

All claims expressed in this article are solely those of the authors and do not necessarily represent those of their affiliated organizations, or those of the publisher, the editors and the reviewers. Any product that may be evaluated in this article, or claim that may be made by its manufacturer, is not guaranteed or endorsed by the publisher.
